# Transmission of *Xanthomonas albilineans* by the spittlebug, *Mahanarva fimbriolata* (Hemiptera: Cercopidae), in Brazil: first report of an insect vector for the causal agent of sugarcane leaf scald

**DOI:** 10.1093/jisesa/iead116

**Published:** 2023-12-18

**Authors:** Andressa Peres Bini, Marina Carnaz Duarte Serra, Izadora Farina Pastore, Carolina Veluci Brondi, Luis Eduardo Aranha Camargo, Claudia Barros Monteiro-Vitorello, Marie-Anne van Sluys, Guilherme Duarte Rossi, Silvana Creste

**Affiliations:** Instituto Agronômico (IAC), Centro de Cana, Ribeirão Preto, SP 14032-800, Brazil; Instituto Agronômico (IAC), Centro de Cana, Ribeirão Preto, SP 14032-800, Brazil; Instituto Agronômico (IAC), Centro de Cana, Ribeirão Preto, SP 14032-800, Brazil; Instituto Agronômico (IAC), Centro de Cana, Ribeirão Preto, SP 14032-800, Brazil; Faculdade de Ciências Agrárias e Veterinárias, Universidade Estadual Paulista (Unesp), Jaboticabal, SP 14884-900, Brazil; Escola Superior de Agricultura ‘Luiz de Queiroz’, Universidade de São Paulo (USP), Piracicaba, SP 13418-900, Brazil; Escola Superior de Agricultura ‘Luiz de Queiroz’, Universidade de São Paulo (USP), Piracicaba, SP 13418-900, Brazil; Departamento de Botânica, Instituto de Biociências, Universidade de São Paulo (USP), São Paulo, SP 05508-090, Brazil; Faculdade de Ciências Agrárias e Veterinárias, Universidade Estadual Paulista (Unesp), Jaboticabal, SP 14884-900, Brazil; Instituto Agronômico (IAC), Centro de Cana, Ribeirão Preto, SP 14032-800, Brazil

**Keywords:** pathogen transmission, plant disease, hemipteran, qPCR diagnostics, insect vector

## Abstract

Leaf scald is a destructive sugarcane disease caused by the bacterium *Xanthomonas albilineans* (Ashby) Dowson. This pathogen presents the gene cluster SPI-1 T3SS, a conserved feature in pathogens vectored by animals. In this study, the competence of *Mahanarva fimbriolata* (Stål), a spittlebug commonly found in sugarcane fields in Brazil, was evaluated for the transmission of *X. albilineans*. Artificial probing assays were conducted to investigate the ability of *M. fimbriolata* adults to acquire *X. albilineans* from artificial diets containing the pathogen with subsequent inoculation of *X. albilineans* into pathogen-free diets. Plant probing assays with *M. fimbriolata* adults were conducted to evaluate the acquisition of *X. albilineans* from diseased source plants and subsequent inoculation of healthy recipient sugarcane plants. The presence of *X. albilineans* DNA in saliva/diet mixtures of the artificial probing assays and both insects and plants of the plant probing assays were checked using TaqMan assays. The artificial probing assays showed that *M. fimbriolata* adults were able to acquire and inoculate *X. albilineans* in diets. Plant probing assays confirmed the competence of *M. fimbriolata* to transmit *X. albilineans* to sugarcane. Over the entire experiment, 42% of the insects had acquired the pathogen and successful inoculation of the pathogen occurred in 18% of the recipient-susceptible sugarcane plants at 72 or 96 h of inoculation access period. Assays evidenced the vector competence of *M. fimbriolata* for transmission of *X. albilineans*, opening new pathways for investigating the biology and the economic impacts of the interaction between *X. albilineans* and *M. fimbriolata*.

## Introduction

Leaf scald is a destructive disease of sugarcane caused by *Xanthomonas albilineans* (Ashby) Dowson. This pathogen is a Gram-negative bacterium of the Gammaproteobacteria class, the Xanthomonadales order, and the Xanthomonadaceae family (Saddler and Bradbury 2005). Many Xanthomonadaceae, especially species from the genera *Xanthomonas*, cause plant diseases (Marguerettaz et al. 2011). *Xanthomonas albilineans* colonizes mainly the xylem but also the nonvascular tissues of sugarcane, with high multiplication rates before any visible symptom (Mensi et al. 2014, Pieretti et al. 2015b). Leaf scald symptoms in sugarcane vary from a single, sharply defined narrow white stripe to complete wilting and necrosis of infected leaves leading to plant death (Birch 2001).

Leaf scald was first recorded in Australia in 1911 and has been reported in at least 66 countries worldwide (Daugrois et al. 2012, Cervantes-Romero et al. 2021). Since then, progress in the ecology and epidemiology of the disease has been made, including studies to understand its dissemination. Transmission of *X. albilineans* to sugarcane plants is currently assumed to occur via infected cutting tools or infected sugarcane cuttings and, under heavy rainfalls, through aerial transmission (Daugrois et al. 2003, Champoiseau et al. 2009, An et al. 2020).

Based on genomic studies, *X. albilineans* has unusual features compared with other xanthomonads, such as a reduced genome and the absence of a Hypersensitive response and pathogenicity-Type III Secretion System (Hrp-T3SS) gene cluster, which is a crucial key component for most *Xanthomonas* spp.-plant interactions (Pieretti et al. 2009, 2012, Zhang et al. 2020, Li et al. 2022). Another intriguing feature of *X. albilineans* is the presence of a *Salmonella* Pathogenicity Island-1 Type III Secretion System (SPI-1 T3SS). The SPI-1 T3SS is primarily found in insect-vectored bacterial pathogens or symbionts and is often necessary for bacterial invasion and persistence in the insect host (Stevens et al. 2002, Egan et al. 2014). For instance, *Pantoea stewartii* (the causal agent of Stewart’s wilt of maize) have two T3SSs: one Hrp-T3SS essential for maize colonization/pathogenesis and one SPI-1 T3SS required for bacterial persistence in the gut of its flea beetle vector (Correa et al. 2012).

In addition, the SPI-1 T3SS of *X. albilineans* is not involved in sugarcane infection, given that inoculation with knockout mutants revealed that this secretion system is not required for *X. albilineans* to colonize the xylem and cause leaf scald symptoms when compared with the wild type (Marguerettaz et al. 2011). The role of the SPI-1 T3SS in *X. albilineans* remains unclear, but it can be hypothesized that this secretion system is required for the interaction of this plant-invading pathogen with an as-yet-unknown animal, including putative insect vector (Marguerettaz et al. 2011, Pieretti et al. 2015b, Zhang et al. 2020).

Hemipteran insects are devastating pests of crops due to their wide host range, rapid reproduction, and ability to act as vectors of numerous plant-infecting pathogens (Perilla-Henao and Casteel 2016). Populations of the sugarcane spittlebug, *Mahanarva fimbriolata* (Stål) (Hemiptera: Cercopidae), have greatly increased and become a serious pest of Brazilian sugarcane fields after the implementation of the green harvest system (harvest of sugarcane without previous burning of the field) (Dinardo-Miranda et al. 2016, Tonelli et al. 2016, Schöbel and Carvalho 2021). Sugarcane spittlebugs are sap-sucking insects that feed on the xylem, the main niche of *X. albilineans*. In Brazil, *M. fimbriolata* has seasonal occurrences from December to March, which is correlated with the higher temperatures and accumulations of rainfall in this period (da Cunha Borges Filho et al. 2019). Moreover, monocots from the Poaceae family, e.g., pasture plantations, are common hosts of both *X. albilineans* (Birch 2001) and *M. fimbriolata* (Schöbel and Carvalho 2021).

The first suspicion of *X. albilineans* transmission by spittlebugs occurred in April 2017 in a nursery plot of a Brazilian sugarcane mill. Disease-free sugarcane seedlings produced by meristem tip culture, with no history of using cutting instruments showed typical leaf scald disease symptoms, including white pencil lines and necrosis of the leaves after an infestation of the nursery by *M. fimbriolata*, and plants tested PCR positive for *X. albilineans* (S. Creste, personal communication). Considering the coexistence of *M. fimbriolata* and *X. albilineans* in sugarcane fields, we hypothesized the competence of *M. fimbriolata* to act as a vector mediating the transmission of *X. albilineans* in sugarcane. To explore this hypothesis, the present work tested the ability of *M. fimbriolata* to acquire and inoculate this pathogen in artificial probing assays. Additionally, we evaluated the vector competence of *M. fimbriolata*, assessing the acquisition and inoculation rates of *X. albilineans* in susceptible sugarcane plants.

Overall, artificial probing assays confirmed the ability of *M. fimbriolata* to acquire and inoculate the pathogen *X. albilineans*. Furthermore, the plant probing assays showed that *M. fimbriolata* acquired *X. albilineans* from infected source plants and inoculated *X. albilineans* to healthy recipient sugarcane plants, indicating the vector competence of *M. fimbriolata* to transmit *X. albilineans* to sugarcane plants. The data presented here constitute an essential step for research on *X. albilineans*.

## Materials and Methods

### Collection of Insects

*Mahanarva fimbriolata* nymphs were collected over the 2019–2020 seasons (February until March) from sugarcane fields with spittlebug infestations history at ‘Instituto Agronômico (IAC), Centro de Cana, Ribeirão Preto, São Paulo, Brazil (21°11ʹ S, 47°48ʹ W).” The nymphs were collected in the field from sugarcane roots using a fine-tip brush, immediately transferred to sugarcane plantlets with exposed roots, and kept in a cooled container for transport to the laboratory. The nymphs were reared until adulthood under controlled conditions (25 ± 2 °C, 12h:12h photoperiod) on healthy sugarcane plantlets produced by tissue culture and indexed free of *X. albilineans* by TaqMan assays (described below). A prescreening of *M. fimbriolata* adults (approximately 10%) indicated the absence of *X. albilineans* (TaqMan assays described below) in the adults used in the experiments of the present work (Supplementary Figure S1).

### Bacterial Isolate and Inoculum Preparation

*Xanthomonas albilineans* isolate Xa11 (Xa11) was obtained from a symptomatic plant in a sugarcane-growing region in Brazil, confirmed as *X. albilineans* by conventional PCR and selected for the present work given its high aggressiveness to sugarcane SP78-4467-susceptible genotype (Tardiani et al. 2014). The isolate Xa11 was grouped with an average nucleotide identity of 99.94% after genome sequencing with the Guadeloupe Island Strain GPE PC73 (Miranda et al. 2023), which belongs to a specific genetic subgroup known as PFGE-B (Pieretti et al. 2012) and serotype 1 (Pieretti et al. 2015a). Xa11 is phylogenetic related to American strains clustering of *X. albilineans* (Miranda et al. 2023). Xa11 was stored at −80 °C in 20% glycerol (v:v), for long-term maintenance at the microorganism’s collection of “Instituto Agronômico (IAC), Centro de Cana, Ribeirão Preto-SP, Brazil (21º11ʹ S, 47º48ʹ W).”

Xa11 was streaked from glycerol stock on Xas solid medium (Davis et al. 1994) and grown at 28 °C for 6 days (Supplementary Figure S2). After, the bacterial colonies were scraped from the solid medium and transferred to the Xas liquid medium (Davis et al. 1994). The Xa11 inoculum (OD_600nm_ = 0.1) was grown for 20 h at 28 °C, 200 rpm, and complete darkness until reaching the early-exponential phase (OD_600nm_ = 1.5). At that point, the culture was transferred to 50-ml tubes and centrifuged (2,000 g; 5 min; 4 °C). The supernatant was discarded, and the bacteria were suspended to a concentration of 10^8^ CFU/ml (OD_600nm_ = 0.3) in a liquid artificial diet (0.7 mM l-glutamine, 0.1 mM l-asparagine, 1 mM sodium citrate, pH 6.4—Killiny and Almeida 2009) for artificial probing assays or PBS (10 mM sodium phosphate, 137 mM NaCl, pH 7.4) for plant probing assays. The suspended bacteria were kept on ice until their use.

### Healthy Sugarcane Plants

The healthy plants used in the present work were produced at a sugarcane tissue culture laboratory and were provided by “Instituto Agronômico (IAC), Centro de Cana, Ribeirão Preto, São Paulo, Brazil (21º11ʹ S, 47º48ʹ W).” Disease-free sugarcane plantlets (healthy plantlets) were produced in vitro from the meristem tip culture of the sugarcane genotype SP78-4467 susceptible to leaf scald. After in vitro shoot multiplication and rooting, the plantlets were indexed free of *X. albilineans* by TaqMan assays (described below), transplanted to 50 cell seedling trays containing Carolina soil substrate (78% Sphagnum, 22% vermiculite, pH 5.5, electrical conductivity 0.4 mS/cm, water holding capacity 350% m/m, density 130 kg/m^3^) (Carolina Soil do Brasil Ltda., Pardinho, SP, Brazil) and kept in a vector-proof greenhouse for 30 days for acclimatization/hardening. At this stage, some plantlets were used for rearing the spittlebugs collected from sugarcane fields. The remaining plantlets were transplanted to 0.3-liter pots containing Carolina soil substrate and cultivated in a vector-proof greenhouse for an additional 2 months until they reached 3 months old for the plant probing assays (Supplementary Figure S3).

### Acquisition and Inoculation of *X. albilineans* by *M. fimbriolata* in Artificial Diets

To test the hypothesis of *M. fimbriolata* being a vector of *X. albilineans*, we first tested in 2019 the ability of this sap-sucking insect to acquire and inoculate the pathogen based on artificial probing assays (Tanne et al. 2001, Esteves et al. 2019, Lenzi et al. 2019). For that, liquid artificial diets (Killiny and Almeida 2009) containing Xa11 (10^8^ CFU/ml) were offered to the *M. fimbriolata* adults through a membrane feeding system (Supplementary Figure S4), as described below. The insects were starved for 1 h and then introduced into the feeding tubes (1 adult per tube), which consisted of clear, translucent, 5-ml microtubes whose caps were removed. The caps were filled with 1 ml of diet containing the bacteria and sealed with a double layer of stretched Parafilm (Parafilm, Bemis, Oshkosh, WI). Tubes were closed and kept under a source of fluorescent light (150 W) to encourage the insects to probe the diet solution containing bacteria through the Parafilm. In total, 42 tubes containing one insect each and an artificial diet with Xa11 were assembled and the insect acquisition access period (AAP) was 24 h under controlled conditions (25 ± 2 °C, 12h:12h photoperiod). Insect mortality was recorded after the AAP, and the dead insects were discarded. Subsequently, the surviving insects were individually placed into new feeding tubes containing artificial diets without Xa11 for an inoculation access period (IAP) until their death, which generally occurred between 24 and 48 h. Subsequently, the dead insects were removed from the feeding tubes and the diet/saliva mixtures (1 ml) were collected in 2-ml microtubes.

The negative controls consisted of 12 tubes containing one insect each and an artificial diet without Xa11. These tubes went through the same procedure as described above. The experiment was performed using a completely randomized design. The diet/saliva mixtures from the treatment (insect + diet with Xa11) were maintained separately (1 ml). Meanwhile, the negative controls and the diets containing Xa11 used during the AAP were bulked (bulks of 2 samples, 2 ml). All diet/saliva mixtures sampled were freeze-dried and stored at −80 °C until DNA extraction and TaqMan assays for detection/quantification of *X. albilineans* (Supplementary Figure S4).

### Acquisition and Inoculation of *X. albilineans* by *M. fimbriolata* in Sugarcane Plants

Greenhouse experiments (season 2020) were conducted to test the acquisition and inoculation of *X. albilineans* by *M. fimbriolata* in plant probing assays. Experiments were performed based on previous insect vector competence studies (Lenzi et al. 2019, Esteves et al. 2019, 2020, Müller et al. 2021). For this, 3-month-old healthy sugarcane plants were inoculated according to the decapitation method at 30 cm from the soil (Rott et al. 1997, Brumbley et al. 2004), using 50 μl of Xa11 inoculum suspended in PBS (10^8^ CFU/ml). These plants composed the source for the acquisition of *X. albilineans* and were maintained in a vector-proof greenhouse for one month. In total, 15 symptomatic plants were obtained and *X. albilineans* infection was confirmed by TaqMan assays (described below), with the number of copies of *ALB1* gene per reaction (400 ng of template DNA) ranging from 1.45 × 10^5^ to 4.30 × 10^6^ (Supplementary Table S2). These 15 infected plants were used for 3 rounds of AAP, according to the emergence of the spittlebug adults reared on healthy sugarcane plants.

Adult insects were starved for 1 h and then were introduced and trapped inside anti-aphid net cages (2 adults per cage) containing one sugarcane source plant infected by *X. albilineans*. In total, 67 adults experienced 96-h AAP. At the end of the AAP, the surviving individuals (53) were transferred to new cages (one insect per cage) containing one recipient healthy plant for an IAP of 24, 72, and 96 h. Subsequently, the surviving insects (45) had the wings removed and the bodies sampled. The recipient plants were maintained in the anti-aphid net cages and greenhouse conditions for an additional 6 days (144 h post-IAP), for systemic infection of *X. albilineans* (Lin et al. 2018). After this period, the whole aerial tissues (leaves + leaf spindle) of each plant were sampled. Insect mortality was recorded after AAP and IAP, and the dead insects were discarded. The plants for which insect mortality post-IAP was recorded were not evaluated to avoid false negatives due to the death of insects.

For the negative controls, 12 insects individually caged which experienced 96-h AAP and 96-h IAP probing sugarcane healthy plants were evaluated using the same procedure described above. The experiment was performed using a completely randomized design. All insects and plants were collected in liquid nitrogen and stored at −80 °C until DNA extraction and TaqMan assays for detection/quantification of *X. albilineans* (Supplementary Figure S5).

### DNA Extraction

The diet/saliva mixtures (1 or 2 ml for bulked samples) were freeze-dried, resuspended in 50 µl of PBS, and submitted to thermal lysis (95 °C; 10 min). Fresh-frozen tissues of spittlebugs (one adult) or sugarcane (aerial tissues) were ground into a fine powder using pestle, mortar, and liquid nitrogen. DNA extraction from diet/saliva mixtures (1 or 2 ml for bulked samples), spittlebugs (one adult), or sugarcane plants (200 mg of aerial tissues) was performed according to the GenElute Plant Genomic DNA Miniprep Kit protocol (Sigma–Aldrich, Burlington, MA, USA). The concentration and integrity of the genomic DNA were assessed using a Nanodrop spectrophotometer (Thermo Fisher Scientific, Wilmington, DE, USA) and 0.8% agarose gel electrophoresis stained with ethidium bromide (1 µg/ml).

### Detection and Quantification of *X. albilineans* in Diet/Saliva Mixtures, Insects, and Plants

Molecular detection of *X. albilineans* in diet/saliva mixtures, spittlebugs, or sugarcane plants was carried out using quantitative real-time PCR (qPCR). TaqMan assays were performed using specific primers and hydrolysis probes developed for *X. albilineans* based on the gene cluster of albicidin toxin biosynthesis corresponding to the *ALB1* gene (Garces et al. 2014). The qPCR reactions were performed using StepOnePlus Real-Time PCR System (Applied Biosystems, Waltham, MA, USA) and GoTaq Probe qPCR Master Mix (Promega, Madison, WI, USA) in a final volume of 20 μl. Each reaction contained 1× GoTaq Probe qPCR Master Mix (Promega), 0.25 µM of the probe, 0.8 µM of each primer, and 400 ng of template DNA (100 ng/μl). Thermal cycling parameters consisted of an initial preheating step for 2 min at 95 °C followed by 40 cycles at 95 °C for 15 s and 60 °C for 1 min. Positive control samples consisted of DNA obtained from leaf-scald symptomatic plants. Negative control samples consisted of DNA obtained from in vitro cultured plants (healthy plants). Nontemplate samples consisting of Milli-Q water instead of DNA were always included. Reactions were performed in technical duplicates.

A standard curve (Supplementary Figure S6) was prepared using technical triplicates based on a 10-fold serial dilution (10^7^ to 10^1^ copies per reaction) of the targeted sequence of *ALB1* cloned into pGEM-T Easy Vector System (Promega) to determine the limits of detection of the TaqMan assays (12.72 ≤ Cq ≤ 32.50, Supplementary Figure S6). Samples within the detection limits of the TaqMan assays had their averages and the standard deviations of the quantification cycle (Cq) calculated using the technical duplicates. The quantification of *X. albilineans* in each sample was based on the calculated averages and was expressed in the number of copies of the target gene (*ALB1*) per reaction (400 ng of template DNA) as previously defined by Bini et al. (2023).

## Results

### Acquisition and Inoculation of X. albilineans by M. fimbriolata Observed in Artificial Probing Assays

The mortality of *M. fimbriolata* spittlebugs post-AAP in the diets containing *X. albilineans* was 12% (5 of 42 insects). After IAP, TaqMan assays revealed that 89% (33 of 37 diets) of diet/saliva mixtures tested positive for *X. albilineans* (Table 1, Supplementary Table S1). In addition, *X. albilineans* was detected in all diet/saliva mixtures used as pathogen source to 24-h AAP (Table 1, Supplementary Table S1).

**Table 1.** Detection and quantification of *X. albilineans* in diet/saliva mixtures of artificial probing assays

Sample	*X. albilineans* detection and quantification (TaqMan)^d^		
	Feeding diet^e^	Cq avg^f^	Copies avg^g^
Diet/saliva mixtures used to AAP^a^	Positive (21/21)	11.49 ± 1.05	3.27 × 10^7^ ± 2.04
Diet/saliva mixtures after IAP^b^	Positive (33/37)	26.56 ± 3.09	1.18 × 10^3^ ± 8.14
Ctrl (-)^c^	Negative (0/5)	N/D	N/D

^a^Artificial diets containing the bacteria used to fed spittlebug adults through a membrane feeding system to 24-h AAP.

^b^Diet/saliva mixtures solution sampled after IAP.

^c^Negative controls consisted of artificial diets without bacteria offered to the spittlebug adults through a membrane feeding system.

^d^Quantitative real-time PCR (qPCR) detection and quantification of *X. albilineans* in artificial diets using TaqMan.

^e^Numbers in parenthesis show the ratio of qPCR positives to total number of diets tested in each treatment.

^f^Average and standard deviation between biological replicates of quantification cycle (Cq) values of positive diet/saliva mixtures assessed by TaqMan in each treatment. Standard curve detection range (*R*^2^ = 0.99, *E* = 97.21%): Cqs average varies between 12.72 (10^7^ copies per reaction) to 32.50 (10^1^ copies per reaction) and N/D = not detected.

^g^Quantification of *X. albilineans* expressed in numbers of copies of bacterial gene (*ALB1*) per reaction (400 ng of template DNA) assessed in positive samples (average and standard deviation between biological replicates).

For the negative controls, the mortality of *M. fimbriolata* spittlebugs post-AAP was 17% (2 of 12 insects) and the pathogen *X. albilineans* was not detected in diet/saliva mixtures tested post-IAP, confirming that the spittlebugs used in the experimentation were initially pathogen-free (Table 1, Supplementary Table S1).

### Acquisition and Inoculation of *X. albilineans* by *M. fimbriolata* Observed in Plant Probing Assays

*Xanthomonas albilineans* was detected in all plants used as the pathogen source at 96-h AAP (Supp. Table S2). The mortality of *M. fimbriolata* spittlebugs during the entire experiment with plants infected by *X. albilineans* was 33% (22 of 67 insects), with mortality rates of *M. fimbriolata* post-AAP of 21% (14 of 67 insects) and post-IAP of 15% (8 of 53 insects). Consequently, 45 adults of *M. fimbriolata* and plant combinations were evaluated for transmission of *X. albilineans* after AAP and IAP. The acquisition rates were 54%, 36%, and 39% for insects collected after 24-, 72-, or 96-h IAP, indicating that over the entire experiment, 42% (19 of 45 insects) of the insects had acquired the pathogen from source plants (Table 2, Supplementary Table S2). *Xanthomonas albilineans* was not detected in recipient plants after 24-h IAP although 54% of the insects had acquired the pathogen (Table 2, Supplementary Table S2). The pathogen was detected in recipient plants at 72- and 96-h IAP, with inoculation rates of, respectively, 100% (pathogen detected in 5 adult insects and 5 inoculated plants) and 43% (pathogen detected in 7 adult insects and in 3 inoculated plants). Over the entire experiment, the successful transmission of the pathogen occurred in 18% (8 of 45 plants) of the recipient plants (Table 2, Supplementary Table S2).

**Table 2.** Detection and quantification of *X. albilineans* in spittlebug insect *M. fimbriolata* and sugarcane plants. Acquisition and inoculation rates were assessed in adults of *M. fimbriolata* and sugarcane plants from the plant probing assays

*X. albilineans* detection and quantification (TaqMan)								
Insect					Sugarcane			
IAP duration^a^	Adults^b^	Acquisition rates	Cq avg^d^	Copies avg^e^	Plants^c^	Inoculation rates	Cq avg^d^	Copies avg^e^
24 h	7/13	54%	30.39 ± 1.72	8.74 × 10^1^ ± 3.21	0/13	0%	N/D	N/D
72 h	5/14	36%	28.36 ± 3.06	3.47 × 10^2^ ± 8.01	5/14	100%	27.64 ± 4.23	5.63 × 10^2^ ± 17.70
96 h	7/18	39%	27.72 ± 3.40	5.36 × 10^2^ ± 10.04	3/18	43%	31.79 ± 0.40	3.37 × 10^1^ ± 1.31
Ctrl (-)^f^	0/8	0%	N/D	N/D	0/8	0%	N/D	N/D

^a^After a 96-h acquisition access period (AAP), the insects were transferred to cages with healthy sugarcane plants for 24-, 72-, and 96-h IAP. After IAP periods, the insects and plants were collected for *X. albilineans* detection and to estimate the acquisition and inoculation rates, respectively.

^b^Number of insects infected/total number tested.

^c^Number of plants infected/total number tested.

^d^Average of quantification cycle (Cq) values of positive insects or plants assessed by TaqMan for each treatment and the standard deviation between biological replicates. Standard curve detection range (*R*^2^ = 0.99, *E* = 97.21%): Cqs average varies between 12.72 (10^7^ copies per reaction) to 32.50 (10^1^ copies per reaction) and N/D = not detected.

^e^Quantification of *X. albilineans* expressed in numbers of copies of bacterial gene (*ALB1*) per reaction (400 ng of template DNA) assessed in positive samples (average and standard deviation between biological replicates);.

^f^Negative controls consisted of insects fed on sugarcane healthy plants that pass through 96-h AAP and 96-h IAP.

For the negative controls, the mortality of *M. fimbriolata* spittlebugs during the entire experiment was 34% (4 of 12 insects), with mortality rates of *M. fimbriolata* post-AAP of 17% (2 of 12 insects) and post-IAP of 20% (2 of 10 insects). *Xanthomonas albilineans* were not detected in the 8 negative controls, including the insects and the plants on which these insects were fed (Table 2, Supplementary Table S2). The lack of detection of *X. albilineans* in insects confirmed that spittlebugs used in our study were initially pathogen free.

**Fig. 1.**
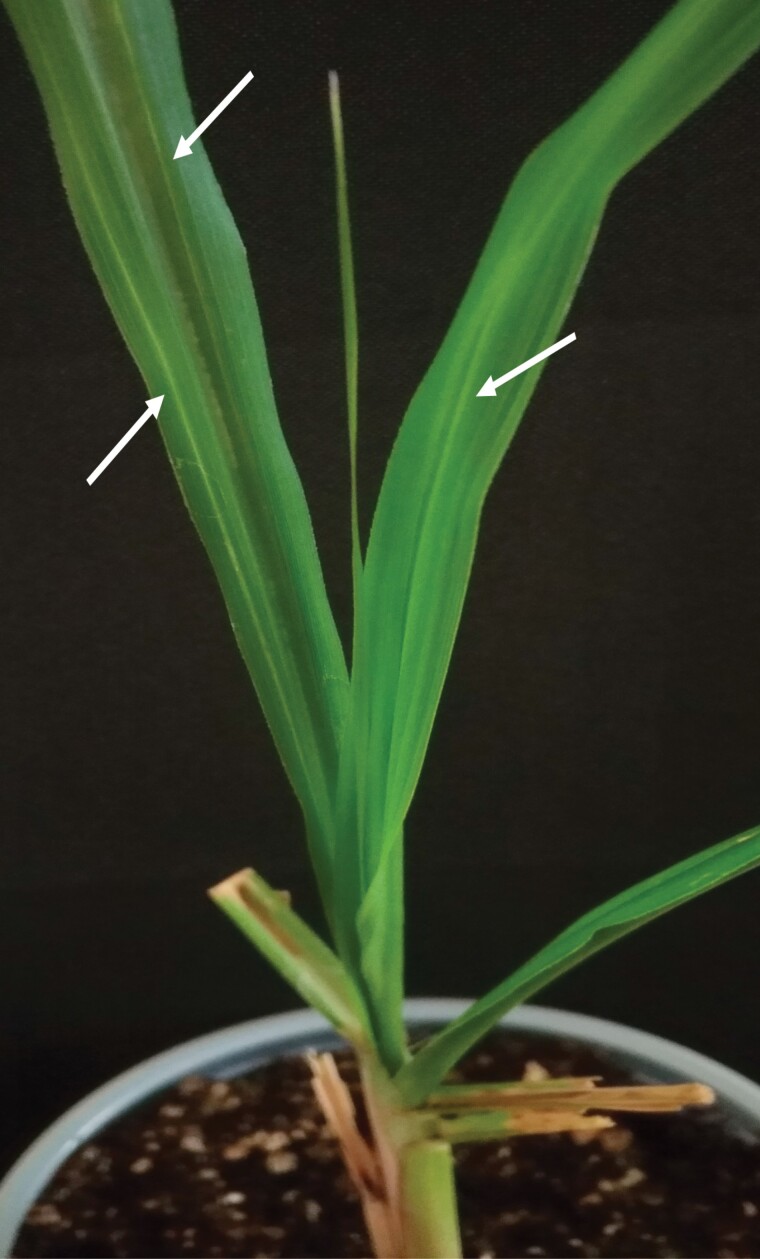
Three-month-old sugarcane plant exhibiting symptoms of leaf scald disease. The white pencil lines (white arrows) were observed in sugarcane leaves 144 h after the 72-h IAP treatment.

Among the 8 recipient plants that tested positive for *X. albilineans*, 5 were obtained for a 72-h IAP and 3 for a 96-h IAP, with *X. albilineans* gene copies ranging from 2.86 × 10^1^ to 1.96 × 10^4^ (Table 3). Plants with the lowest amounts of *X. albilineans* gene copies (10^1^ copies per reaction) were obtained from 72- and 96-h IAPs and showed no symptoms of sugarcane leaf scald (Table 3). On the other hand, 3 plants with the highest amounts of *X. albilineans* gene copies (10^2^, 10^3^, and 10^4^ copies per reaction) were obtained from 72-h IAP (Table 3) and exhibited symptoms of the disease (leaves with white pencil-line streaks) at 144 h after the 72-h IAP (Fig. 1).

**Table 3.** Recipient sugarcane plants that tested positive for *X. albilineans* after being exposed to spittlebug insect *M. fimbriolata* that fed on source plants infected with the pathogen

Plant^a^	*X. albilineans* detection and quantification by TaqMan^b^				Symptoms^e^
	Plants	IAP	Cq avg^c^	Copies avg^d^	
P19	Positive	72 h	23.93 ± 0.02	7.02 × 10^3^ ± 1.02	Present
P20	Positive	72 h	29.20 ± 0.04	1.95 × 10^2^ ± 1.02	Present
P21	Positive	72 h	22.42 ± 0.13	1.96 × 10^4^ ± 1.10	Present
P26	Positive	72 h	31.98 ± 0.27	2.96 × 10^1^ ± 1.20	Absent
P27	Positive	72 h	30.69 ± 0.03	7.11 × 10^1^ ± 1.02	Absent
P28	Positive	96 h	32.03 ± 0.03	2.86 × 10^1^ ± 1.02	Absent
P40	Positive	96 h	32.01 ± 0.00	2.91 × 10^1^ ± 1.00	Absent
P62	Positive	96 h	31.33 ± 0.86	4.59 × 10^1^ ± 1.79	Absent

^a^Sugarcane plants identification (Supplementary Table S2).

^b^Quantitative real-time PCR (qPCR) detection and quantification of *X. albilineans* in plants using TaqMan after an IAP of 72 and 96 h.

^c^Average of quantification cycle (Cq) values of positive plants assessed by TaqMan for each treatment and the standard deviation between 2 technical replicates. Standard curve detection range (*R*^2^ = 0.99, *E* = 97.21%): Cqs average varies between 12.72 (10^7^ copies per reaction) to 32.50 (10^1^ copies per reaction).

^d^Quantification of *X. albilineans* expressed in numbers of copies of bacterial gene (*ALB1*) per reaction (400 ng of template DNA) assessed in positive samples (average and standard deviation between 2 technical replicates).

^e^Leaf scald disease symptoms evaluated at 144 h after IAP.

## Discussion

The results of the present study provide new information about the transmission of *X. albilineans* by *M. fimbriolata* in sugarcane, unveiling the contribution of this spittlebug species to pathogen spread in addition to infected cutting tools, infected stalk cuttings taken from nonsymptomatic plants and aerial transmissions already reported (Daugrois et al. 2003, Champoiseau et al. 2009). The observed leaf scald transmission by *M. fimbriolata* corroborates the hypothesis of an insect vector for *X. albilineans* mounted by Pieretti et al. (2009, 2015b) based on the presence of an SPI-1 T3SS, a secretion system not related to plant pathogenicity but probably related with colonization and persistence of the pathogen in intermediary insect vectors (Nadarasah and Stavrinides 2011, Correa et al. 2012).

No previous studies established *X. albilineans* transmission by an insect. Nonetheless, Marguerettaz et al. (2011) reported a PCR screening for the presence of *X. albilineans* in insects collected in sugarcane fields in Guadeloupe that failed to identify any insect host for this sugarcane pathogen (M. Royer, unpublished data). In the present study, we observed the ability of the spittlebug *M. fimbriolata*, to acquire and inoculate *X. albilineans* in artificial diets. Artificial probing assays represent a well-established method (Tanne et al. 2001, Esteves et al. 2019, Lenzi et al. 2019), which can be useful for screening other potential insect vectors that will contribute to the understanding of the transmission of *X. albilineans*, including other non-xylem specialists associated with sugarcane and also other xylem-feeding *Mahanarva* species, *M. posticata* (Stål), *M. spectabilis* (Distant), and *M. liturata* (Le Peletier and Serville) known for damaging sugarcane (Schöbel and Carvalho 2020, 2021).

Using plant probing assays, the bacterium *X. albilineans* was detected in the spittlebug *M. fimbriolata* after 24-, 48-, and 96-h IAPs, which was the first positive step for putative transmission. However, the insect did not inoculate the pathogen in recipient plants at 24-h IAP. Possibly, *X. albilineans* requires this retention period within the vector for altering its metabolism to adjust to the host switch (plant host to insect host) before being able to be transmitted (Killiny and Almeida 2014).

The transmission of *X. albilineans* to plants was observed after 72- and 96-h IAP. A higher number of positive plants were detected after 72-h IAP with some of the plants presenting the higher amounts of *X. albilineans* and leaf scald symptoms. The variation in inoculation of the pathogen in the recipient plants after 72- and 96-h IAP may be associated with high variability in transmission ability among adult insect vectors (Tanne et al. 2001). Another possibility is the death of *X. albilineans* after prolonged contact with the insect vector. *Mahanarva fimbriolata* adults are known for injecting a toxic saliva that results in plant cell death (burning of sugarcane symptom—Simões et al. 2016), and this saliva may also be acting as an antibiotic to *X. albilineans*. Therefore, further studies are needed evaluating a larger number of adults and nymphs of *M. fimbriolata* and longer periods for disease development for better conclusions and estimation of transmission rates.

The interaction between *X. albilineans* and *M. fimbriolata* may play an important role in the dissemination of this pathogen in Brazil. *Mahanarva fimbriolata* is an insect commonly found in sugarcane and pasture plantations (Schöbel and Carvalho 2021), which may also host *X. albilineans* (Birch 2001). *Mahanarva fimbriolata* is the most common and damaging spittlebug of sugarcane, being reported in 19 of 26 states of Brazil (Schöbel and Carvalho 2021), with high abundance in São Paulo state (14.60 spittlebugs per m of furrow), the most important sugarcane production area of the country (da Cunha Borges Filho et al. 2019).

The seasonal occurrence of *M. fimbriolata* in Brazil is clearly correlated with higher temperatures, particularly when combined with higher rainfall during summer (da Cunha Borges Filho et al. 2019). Similar weather conditions were correlated with outbreaks of leaf scald disease via aerial transmission (Champoiseau et al. 2009, Daugrois et al. 2012). The wide geographical distribution of *M. fimbriolata* and the favoring seasonal environmental conditions for the development of both *M. fimbriolata* and *X. albilineans* need further investigation since the insect may also favor the dissemination of the disease in the summer seasons in Brazil.

To our knowledge, this is the first report demonstrating *M. fimbriolata* adults as a competent vector of *X. albilineans* in sugarcane-susceptible plants. However, it is essential to emphasize that the insect’s ability to transmit the pathogen depends on its interactions with the bacterial isolate and host plant in the field (Lopes et al. 2009). Future transmission studies involving other time points, insect species, isolates of *X. albilineans*, sugarcane genotypes with different levels of resistance to leaf scald, as well as if the insect transmission can affect the productivity of sugarcane are questions raised from the present study and should be further investigated for better comprehension of vector importance in leaf scald epidemiology.

## Supplementary Material

iead116_suppl_Supplementary_Figures_S1-S6

iead116_suppl_Supplementary_Tables_S1-S2
