# Supplementary material for: Transmission of Xanthomonas albilineans by the spittlebug, Mahanarva fimbriolata (Hemiptera: Cercopidae), in Brazil: first report of an insect vector for the causal agent of sugarcane leaf scald
Source: J Insect Sci. 2023 Dec 18;23(6):28. doi: 10.1093/jisesa/iead116 (PMC10727476; doi:10.1093/jisesa/iead116)
Supplement: iead116_suppl_Supplementary_Figures_S1-S6 [file iead116_suppl_supplementary_figures_s1-s6.pdf]

Journal of Insect Science - Supporting Information - Figures S1, S2, S3, S4, S5, S6

Article title:

Transmission of *Xanthomonas albilineans* by the spittlebug *Mahanarva fimbriolata* (Hemiptera: Cercopidae) in Brazil: First report of an insect vector for the causal agent of sugarcane leaf scald.

The following Supporting Information is available for this article:

Figure S1. Insects used in the present study.

Figure S2. Bacterial isolate used in the present study.

Figure S3. Disease-free sugarcane plantlets (healthy plantlets) used in the present study.

Figure S4. Artificial probing assays and *Xanthomonas albilineans* detection/quantification.

Figure S5. Plant probing assays and *Xanthomonas albilineans* detection/quantification.

Figure S6. Standard curve used to determine the limits of detection and to quantify *Xanthomonas albilineans* in the present study.

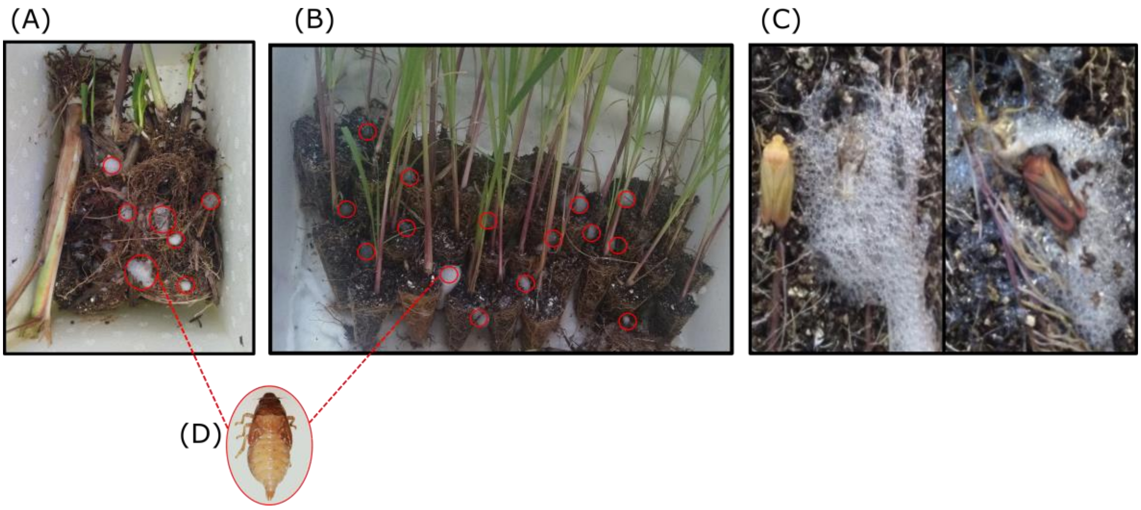

Figure S1. Insects used in the present study. *Mahanarva fimbriolata* nymphs were collected from sugarcane roots in field (A) and transferred to disease free sugarcane plantlets (healthy sugarcanes) with roots exposed (B). The nymphs were maintained in laboratory conditions until fully winged adults emergence (C). Red circles indicate the nymphs (D), that have the habit to develop inside a self-produced foam nest.

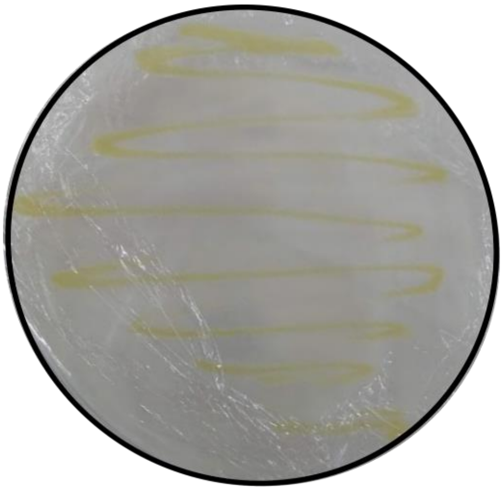

Figure S2. Bacterial isolate used in the present study. *Xanthomonas albilineans* isolate Xa11 streaked from glycerol stock and cultivated in Xas solid medium at 28ºC for 6 days (Davis et al., 1994). The isolate is part of the microorganism's collection of "Instituto Agronômico (IAC), Centro de Cana, Ribeirão Preto-SP, Brazil."

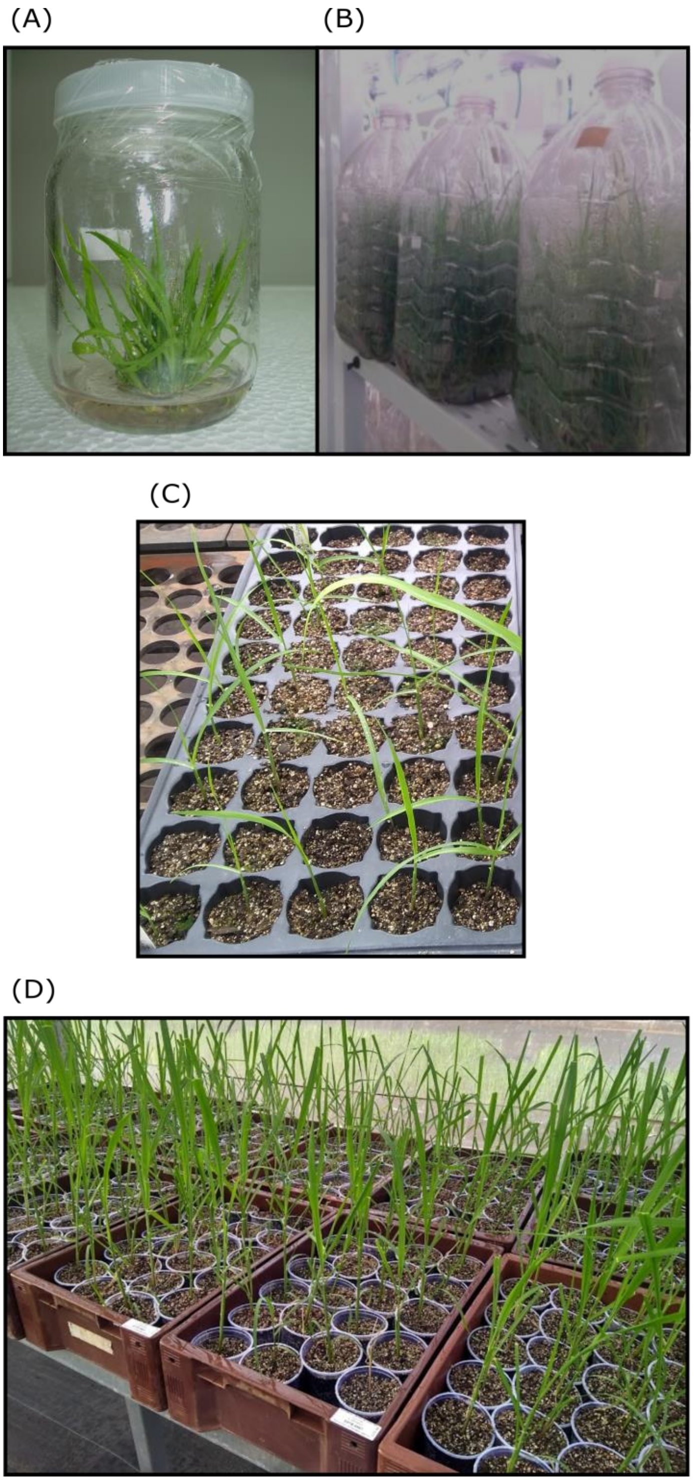

Figure S3. Disease free sugarcane plantlets (healthy plantlets) used in the present study. Sugarcane plantlets produced *in vitro* from meristem tips of SP78-4467 susceptible genotype (shoot multiplication and rooting A, B). The plantlets were transplanted to 50 cell seedling trays for acclimatization/hardening (C) and were kept in a vector-proof greenhouse for 30 days. After, the plantlets were transplanted to 0.3-liter pots (D) and were cultivated in a vector-proof greenhouse until their reach 3-month-old for the plant probing assays.

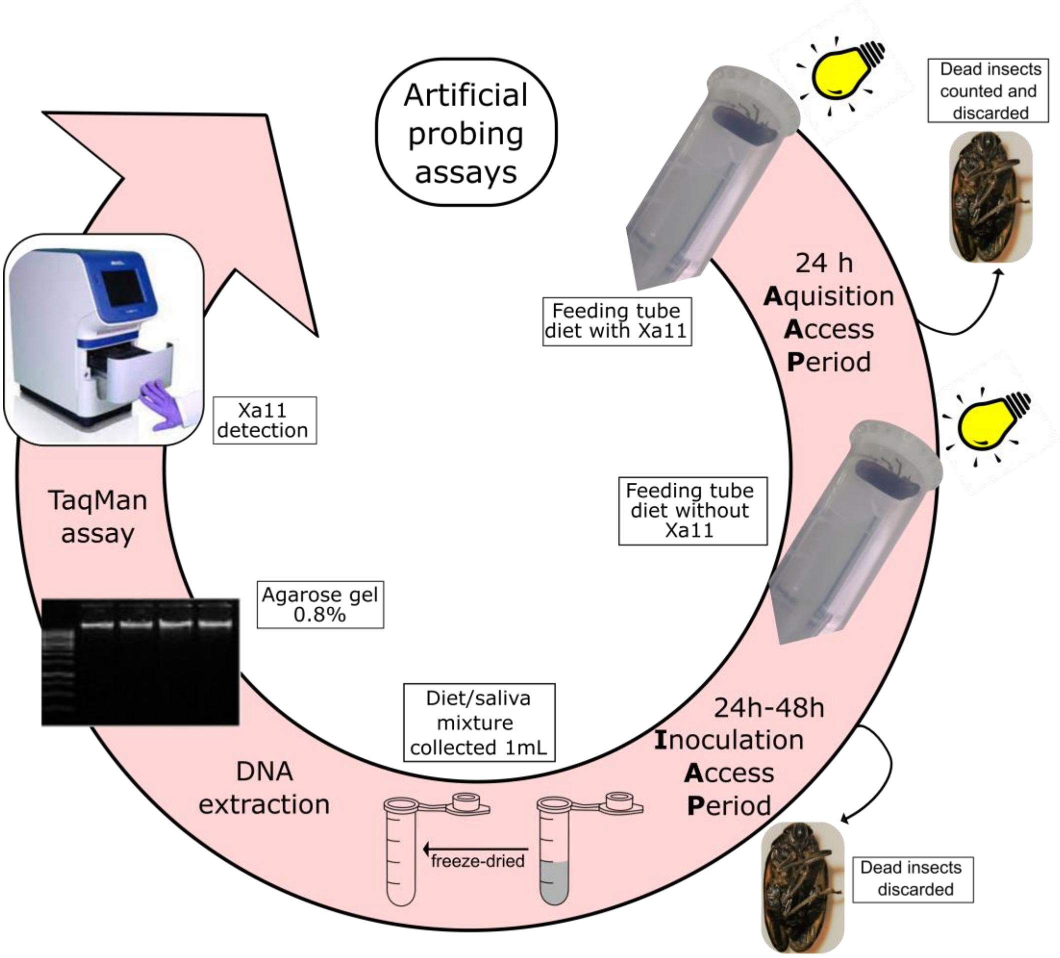

Figure S4. Artificial probing assays and *Xanthomonas albilineans* detection/quantification. A membrane feeding tube system containing Xa11 was assembled with caps tubes facing a source of light to encourage the insects to probe the diet solution containing the bacteria. The insects were allowed an acquisition access period (AAP) of 24 h. After AAP, surviving individuals were placed into a new tube containing a diet without bacteria for an inoculation access period (IAP) until their death (24 h to 48 h). The insects were discarded and the diet/saliva mixtures were collected, freeze-dried, and stored at -80°C until DNA extraction and TaqMan assays for detection/quantification of *X. albilineans.*

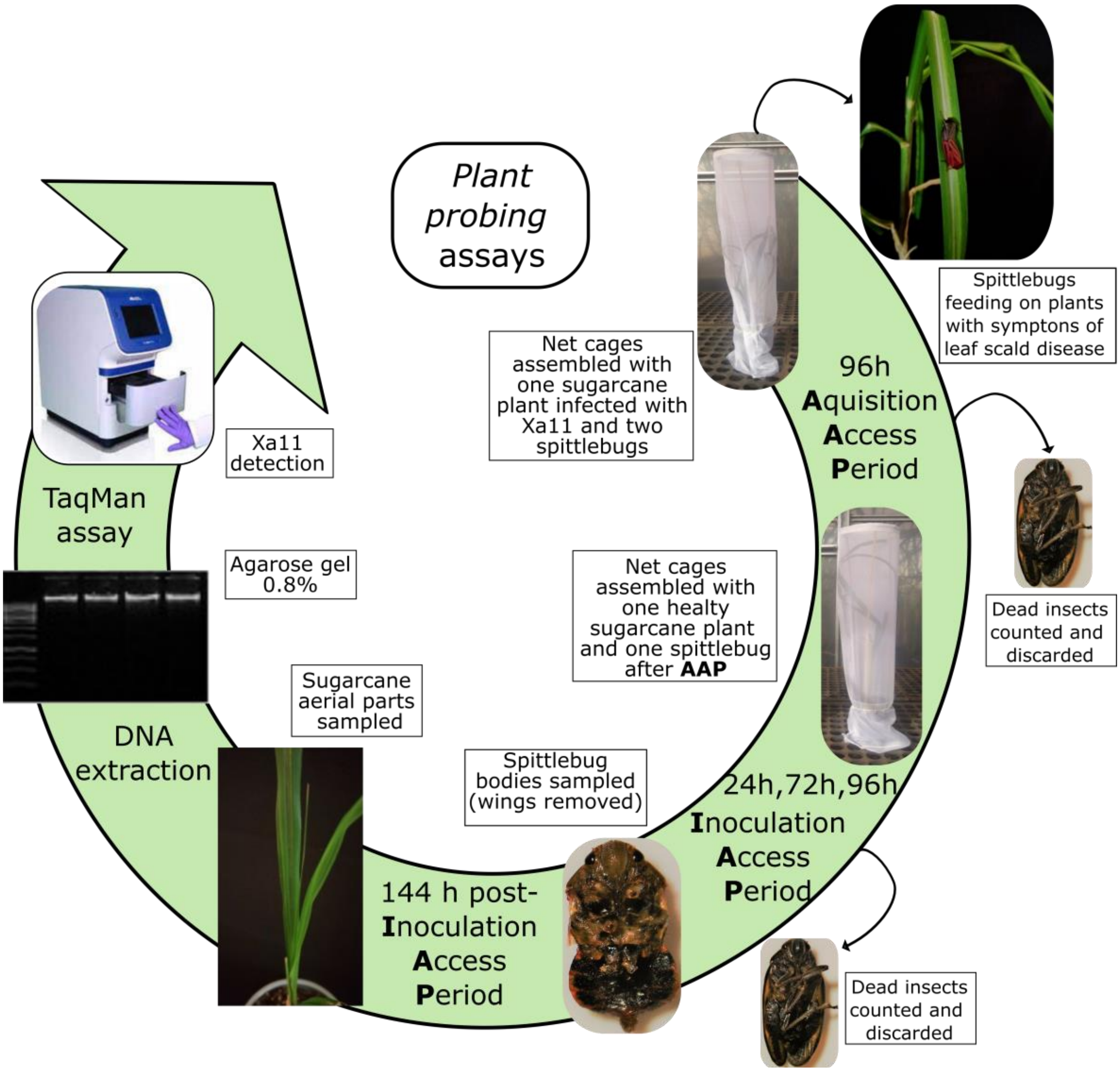

Figure S5. Plant probing assays and *Xanthomonas albilineans* detection/quantification. Two sugarcane spittlebugs were introduced in anti-aphid net cages containing one sugarcane source plant infected by the bacteria with leaf scald symptoms for an acquisition access period (AAP) of 96 h. After AAP, the surviving individuals were placed in new cages containing healthy recipient plants (free of bacteria) for inoculation access period (IAP) of 24 h, 72 h and 96 h. After IAP, the surviving insects had the wings removed and the bodies sampled. The plants were maintained in the anti-aphid net cages and in greenhouse conditions for additional 6 days (144 h post-IAP) until its whole aerial tissues (leaves + leaf spindle) were sampled. Insects and plants were collected in liquid nitrogen and stored at -80°C until DNA extraction and TaqMan assays for detection/quantification of *X. albilineans*.

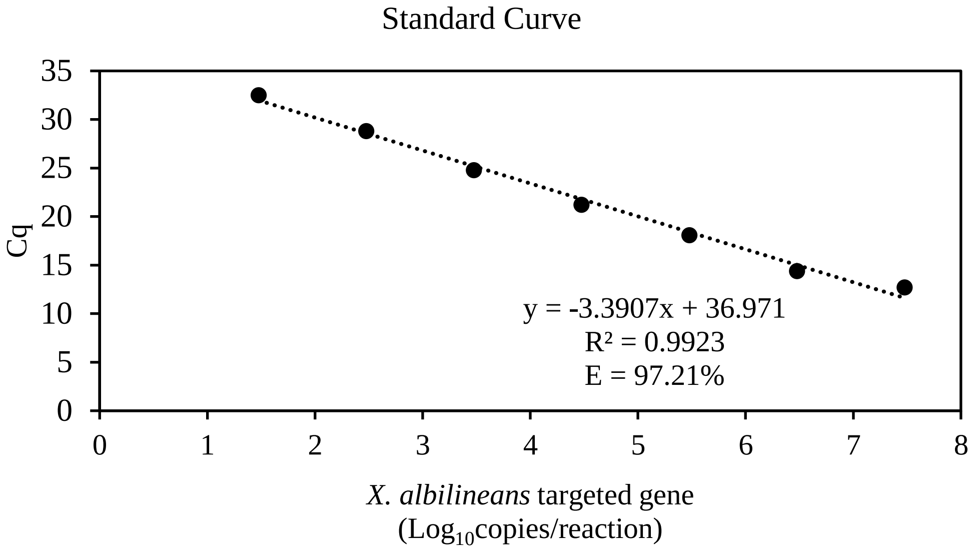

Figure S6. Standard curve used to determine the limits of detection and to quantify *Xanthomonas albilineans* in the present study. Standard curve (Standard dilution series) was obtained by plotting the number of copies of targeted gene (*ALB1*) per reaction versus the quantification cycle (Cq)* detected by qPCR. *Average of the Cq values obtained for three technical replicates for each dilution. The standard error is too low to be visible in the graph.
